# Select Cover Crop Residue and Soil Microbiomes Contribute to Suppression of Fusarium Root and Crown Rot in Barley and Soybean

**DOI:** 10.3390/microorganisms12020404

**Published:** 2024-02-17

**Authors:** Harini S. Aiyer, Andrew McKenzie-Gopsill, Aaron Mills, Adam John Foster

**Affiliations:** 1 Agassiz Research and Development Center, Agriculture and Agri-Food Canada, Agassiz, BC V0M 1A2, Canada; 2 Faculty of Land and Food Systems, The University of British Columbia, Vancouver, BC V6T 1Z4, Canada; 3 Charlottetown Research and Development Center, Agriculture and Agri-Food Canada, Charlottetown, PE C1A 4N6, Canada

**Keywords:** Fusarium root and crown rot (FRCR), cover crops, soil microbiome, disease-suppressive soil, *Fusarium cerealis*, *Fusarium oxysporum* species complex, *Fusarium commune*, sorghum-sudangrass suppresses *Fusarium*

## Abstract

Fusarium root and crown rot (FRCR) negatively impact several economically important plant species. Cover crops host different soil and residue microbiomes, thereby potentially influencing pathogen load and disease severity. The carryover effect of cover crops on FRCR in barley and soybean was investigated. Field trials were conducted in Prince Edward Island, Canada. Two cover crops from each plant group, including forbs, brassicas, legumes, and grasses, were grown in a randomized complete block design with barley and soybean planted in split plots the following year. Barley and soybean roots were assessed for FRCR through visual disease rating and *Fusarium* spp. were isolated from diseased tissue. Fungal and bacterial communities in cover crop residues were quantified using amplicon sequencing. The disease-suppressive effects of soil were tested in greenhouse studies. The results indicated that sorghum-sudangrass-associated microbiomes suppress *Fusarium* spp., leading to reduced FRCR in both barley and soybean. The oilseed radish microbiome had the opposite effect, consequently increasing FRCR incidence in barley and soybean. The results from this study indicate that cover crop residue and the associated soil microbiome influence the incidence and severity of FRCR in subsequent crops. This information can be used to determine cover cropping strategies in barley and soybean production systems.

## 1. Introduction

Fusarium root and crown rot (FRCR) impact a wide range of economically important crops by causing significant a decrease in crop quality and yield [1,2]. *Fusarium* resting structures are known to survive harsh winter conditions in soil and crop residues, potentially increasing the susceptibility of subsequently planted crops to FRCR, especially under no-till cropping systems [3,4,5]. As such, disease management strategies must involve early preventative measures, such as the use of fungicide seed treatments, to allow crops to establish without being affected by the pathogen early in the growing season [6]. However, severe weather conditions, such as drought, are known to increase plant susceptibility to FRCR, and fungicide treatments may not be as effective under these conditions [7]. As climate change increases the frequency of such extreme weather events, conservation agriculture practices must be adopted to ensure sustainable and cost-efficient crop production [8,9]. One such practice is the use of cover crops to increase diversity in crop rotation, as well as to restore soil nutrients and organic matter. Increased cropping diversity is expected to break disease cycles by including non-host crops in the rotation, which reduces the pathogen load through competition for resources, as well as through the secretion of anti-microbial compounds in root exudates [10,11].

Crop rotations including diverse crop groups are encouraged in Prince Edward Island (PE), Canada to reduce soil erosion and to increase soil organic matter [12]. However, despite the many benefits, growers may be reluctant to include cover crops in crop rotations because of the lack of immediate profits associated with not harvesting the crop in that growing season. As such, choosing the cover crop that minimizes loss and potentially contributes to better productivity in subsequent years becomes critical. The cover crop identity within a rotation can have a significant influence on the soil microbial community composition [11,13]. For example, an increase in the abundance of plant pathogenic fungi has been reported following crops such as oilseed radish (*Raphanus sativus* L.), alfalfa (*Medicago sativa* L.), and phacelia (*Phacelia tanacetifolia* Benth.) [13]. Bainard et al. [14], found that the sequential planting of pulse crops can increase the population of certain soil-borne pathogens, including several *Fusarium* spp.. Buckwheat (*Fagopyrum esculentum* Moench.), which has been shown to increase phosphorus availability in the soil and to suppress diseases caused by pathogenic fungi *Rhizoctonia solani* Kühn [teleomorph: *Thanatephorus cucumeris* (Frank) Donk] and oomycete *Pythium* spp. Trow [15,16]. Noronha [17] has also shown that planting brown mustard (*Brassica juncea* L.) and buckwheat as cover crops reduces damage by wireworms (Coleoptera: *Elateridae*) in subsequent potato (*Solanum tuberosum* L.) crops, subsequently preventing the secondary infection of damaged tissue by pathogenic microorganisms. Crop rotation can also promote disease-suppressive soils by increasing the abundance of antagonistic microorganisms [5,18]. The choice of cover crop may influence the disease-suppressive potential of the soil environment, with crops such as sorghum-sudangrass (*Sorghum × drummondii* (Nees ex. Steud.) Millsp. and Chase) and buckwheat increasing the abundance of certain beneficial fungal taxa in the soil, such as symbiotrophic fungi and mycoparasitic fungi such as *Clonostachys* spp. ((Link) Schroers; synonym: *Gliocladium roseum*) and *Trichoderma* spp. (Pers.) [13]. Buckwheat and sorghum-sudangrass are also known to suppress weeds, potentially through their allelopathic effects [19]. As more information is generated on the effects of integrating cover crops into modern rotations, there is a need for more information on the dynamics of pathogen levels in these systems. Understanding the relationship between disease-suppressive soil microbiomes associated with cover crops and FRCR in subsequently planted crops is of great interest [5]. In this study, representative cover crops from four major crop groups, including brassicas, legumes, forbs, and grasses, were used to better understand the relationship between *Fusarium* abundance in crop residue and FRCR incidence in subsequently planted barley (*Hordeum vulgare* L.) and soybean (*Glycine max* (L.) Merr).

On average, barley and soybean are amongst the two highest acreage crops in Atlantic Canada. PE is the largest producer of these cash crops in this region, having produced 89,074 metric tons of barley and 44,819 metric tons of soybean in 2021 [20]. Both of these crops are susceptible to FRCR, with several known causal agents including *Fusarium oxysporum* ((Schlecht.) emend. Snyder and Hansen), *F. graminearum* (Schwabe; teleomorph: *Gibberella zeae* (Schweinitz) Petch.), *F. sporotrichioides* ((Sherb.) Bilai), *F. cerealis* ((Cooke) Sacc.; synonym: *F. crookwellense* (Burgess) Nelson and Toussoun), *F. equiseti* ((Corda) Sacc.), *F. pseudograminearum* (O’Donnell and Aoki; teleomorph: *Gibberella coronicola*), and *F. culmorum* ((Wm.G.Sm.) Sacc. [21,22,23,24,25,26,27]. FRCR can be difficult to detect as the pathogens may infect the crop, reducing water and nutrient uptake without causing obvious visible symptoms [5]. As such, it is important that we understand how cover crops used in rotation with barley and soybean affect *Fusarium* load in the soil and residue and, more importantly, how they affect FRCR incidence and severity.

The primary aim of this study was to assess the effects of selected cover crops on the *Fusarium* abundance in crop residue, and how that affects FRCR in subsequently planted barley and soybean. Furthermore, the potential disease-suppressive effects of certain cover crop soils were also tested under high *Fusarium* pressure. Our objectives were to test whether (1) the choice of cover crops will influence FRCR in barley and soybean in the subsequent year; whether (2) FRCR will positively correlate with pathogen load in the residue; and whether (3) barley grown in soil with an increased abundance of beneficial microbial taxa will have lower rates of FRCR. This work is adapted from the MSc thesis of Harini Aiyer, who is one of the co-authors for this paper [28].

## 2. Materials and Methods

### 2.1. Field Trial Set Up

Effects of cover crops on FRCR in barley and soybean were studied at Agriculture Agri-Food Canada Harrington Research Farm (46°20′47.4″ N 63°10′25.5″ W; PE, Canada). Field trial set-up and microbiome analysis were described previously by Aiyer et al. [13]. Selected cover crops included oilseed radish, brown mustard, alfalfa, crimson clover (*Trifolium incarnatum* L.), buckwheat, phacelia, annual ryegrass (*Lolium multiflorum* Lam.), sorghum-sudangrass, and an unmanaged fallow control. Cover crops were planted in a randomized complete block design with three replicates in 2 m × 10 m plots. The cover crops grew through the season, and were flail-mowed in the autumn each growing season. All leaf litter was left as ground cover during the winter months. In the subsequent year, barley and soybean were direct seeded into each half of the cover crop plots (2 m × 3 m, with 2 m break separating the split plots). Commercially available certified seed was used for all trials. AAC Synergy barley [29] was planted in both trials at a seeding rate of 300 seeds m^−2^. DH401 soybean (Sevita International) was used for the first trial and 25-10 RY soybean (Dekalb) was used in the second trial, as DH401 was not available. All soybean plots were direct seeded at a rate of 55 seeds m^−2^. All commercial seed was treated with Vitaflo 280 (Chemtura, CT, USA) before planting. Seeds were treated to minimize any impact of seed-borne diseases, ensuring that most of the observed symptoms were caused by soil-borne microorganisms.

Cover crop residue was collected in the second year of each trial, before planting barley or soybean [13]. Plant residue was collected from five random locations in each plot and pooled in plastic bags to give approximately 500 mL of composite sample per plot. The plant residue samples were then subsampled into 50 mL conical tubes and stored at −80 °C until further processing.

### 2.2. Metagenomic Analysis

Residue samples were first lyophilized at approximately 100 μbar (VirTis Freezemobile 12ES Freeze Dryer, SP Scientific, NY, USA), for at least 72 h, to remove moisture and ease tissue disruption. The dried samples were then finely ground, and 20 mg was used for DNA extraction with the DNeasy Plant Mini kit (Qiagen, Germantown, MD, USA). DNA concentrations were normalized to approximately 10 ng μL^−1^ and amplicon sequencing of the ITS1 region was conducted using Illumina MiSeq (Genome Quebec, Montreal, QC, Canada) to study fungal communities, and of the 16S rRNA gene using PacBio Sequel (Integrated Microbiome Resource, Halifax, NS, Canada) to study the bacterial communities in the residue. All methods have been previously described for soil samples in Aiyer et al. [13].

Differential abundance analysis was conducted using a negative binomial general linear model (GLM) to find changes in microbial abundance in response to different cover crops. Bioinformatics analysis, including quality trimming and chimera screening for amplicon sequencing results, was carried out using CLC (Qiagen, Germantown, MD, USA) [13]. Sequences with 97% similarity were grouped into operational taxonomic units (OTUs), which were assigned taxonomic classifications based on the UNITE dynamic reference database version 8 (4 February 2020) [30] for fungi and the SILVA database version 132 SSU Ref NR 99 [31] for bacteria. Likelihood ratio test was used to test significance of OTU by cover crop. Trophic groups for fungal OTUs were assigned using FUNGuild [32]. Bacterial functional groups were assigned using FAPROTAX [33]. The results were visualized in a heatmap created using a fold change comparison of the OTUs present in the cover crop residue compared to the fallow residue.

### 2.3. Field Disease Assessment

Barley and soybean were destructively sampled in randomly selected 30 cm rows from each plot for disease assessment. The effects of cover crops on FRCR of barley and soybean in the subsequent year were assessed through visual root disease rating. Roots were washed to remove soil before disease rating using a modified approach from Chekali et al. [34] and Ellis et al. [35]. The scale ranged between 0: no visual symptoms; 1: discoloration of 1–25% of the root and crown; 2: 26–50% discoloration; 3: 51–75% discoloration; 4: 76–99% discoloration; and 5: 100% discoloration or plant death. Disease severity index was calculated according to Chiang et al. [36] (Equation (1)).

(1) $$DSI\%=\frac{\sum \left(class\;frequency\times score\;of\;rating\;class\right)}{total\;number\;of\;plants\times maximal\;disease\;index}\times 100$$

Twelve randomly selected barley and soybean plants from the first trial and one barley and soybean plant per plot from the second trial were saved for pathogen isolation. Tissue samples were surface-sterilized by first washing in 10% bleach, followed by sterile water then 70% ethanol, before a final rinse with sterile water. Samples were then plated on potato dextrose agar (PDA) media amended with tetracycline [100 μg mL^−1^] and cefotaxime [100 μg mL^−1^] and incubated at room temperature. Approximately two days after initial plating, individual isolates were transferred onto fresh PDA plates amended with the same antibiotics to obtain pure cultures. After 2–3 days of growth, isolates were categorized morphologically as *Fusarium* spp. according to descriptions from Leslie and Summerell [37]; molecular identification was conducted as described below.

### 2.4. Identification of Pathogenic Fusarium *spp.*

Molecular identification of root isolates was carried out using universal primers for barcoding genes, including internal transcribed spacer region (ITS) for all samples and translational elongation factor (Tef-1α) for suspected *Fusarium* isolates (Table 1).

The template DNA preparation for conventional PCR included transferring some mycelium from the pure culture to a 2 mL microcentrifuge tube containing 200 μL AE buffer from the Plant Mini DNeasy kit (Qiagen, Germantown, MD, USA) and silicon dioxide beads. The samples were microwaved for 30 s then ground twice using a Bead Mill 24 Homogenizer (Fisherbrand, Pittsburgh, PA, USA) at speed 6 for 15 s with 5 s pause in between cycles [28]. Conventional PCR was then conducted using 4 μL of this suspension as template along with 20 μL of 2x Phusion High-Fidelity Standard master mix (ThermoFisher), 2 μL of each primer, and 12 μL of molecular grade water per reaction. Reactions were conducted in the SimpliAmp thermal cycler (ThermoFisher) (Table 1). Amplification was confirmed through agarose (1%) gel electrophoresis using 4 μL of PCR product. The remaining 36 μL of PCR product was purified using the MinElute PCR Purification Kit (Qiagen, Germantown, MD, USA) according to manufacturer recommendations, with an elution volume of 30 μL. An aliquot of 10 μL of product was sent for Sanger sequencing (Eurofins Genomics, Toronto, ON, Canada). Ambiguous nucleotides were trimmed from sequences before running NCBI BLASTN to match them to reference sequences in the NCBI standard (nr/nt) database for Fungi [40]. Representative Tef-1α sequences from *Fusarium* spp. isolated from root tissue were chosen for the phylogenetic tree. Sequences were aligned using the multiple sequence comparison by log-expectation (MUSCLE) and trimmed to 500 bp in order to remove ambiguous bases and to normalize the sequence length [41]. The tree was built using the Jukes–Cantor Neighbour joining method with 1000 bootstrap replicates. One sequence identified as *Trichoderma* spp. was used as the outgroup comparison.

### 2.5. Greenhouse Trial

Koch’s postulates are a set of criteria to establish a causal relationship between microbial pathogen and disease [42]. To satisfy Koch’s postulates for FRCR, a selection of isolated *Fusarium* spp. were tested under greenhouse conditions for pathogenicity against soybean and barley with methods slightly modified from Zhou et al. [43]. *Fusarium* inoculum for high pathogen load treatments were prepared on PDA media on standard 90 mm Petri dishes, overlain with sterilized Whatman #1 (85mm) filter papers. *Fusarium* cultures were grown on the filter paper for 7 days until they were approximately 70 mm in diameter. Filter papers with the mycelium were placed in pots filled with Pro-Mix BX growing media (Premier Tech, Rivière-du-Loup, QC, Canada) and covered with an approximately 2 cm layer of potting mix. Sterile filter paper was used as the non-inoculated control. At 21 days after planting, disease was rated using the same disease-rating scale described above.

The effects of certain cover crop soils on FRCR in barley (AAC Synergy) under high and low pathogen load were tested through greenhouse trials. The same trial was attempted with soybean but disease establishment similar to the field did not occur. Soil was collected from cover crop plots in November 2020, after the cover crops were flail-mowed. Treatments included soil collected after growing brown mustard, alfalfa, phacelia, sorghum-sudangrass, and buckwheat, as well as soil collected from a managed weed-free plot referred to here as “no-crop”, as well as autoclaved field soil. Pots were set up in a randomized complete block design with four replicates. Each replicate included seven cover crop treatments in duplicate, which were inoculated with *Fusarium* spp. or plain filter paper. Five seeds were planted per pot approximately 1 cm above filter paper. Drip irrigation was used to maintain consistent moisture levels throughout experiments. Plants were fertilized one week after emergence until flowering, with 30 mL of 1% solution 20:20:20 N:P:K according to manufacturer recommendations.

After emergence, pots were thinned to 1 plant per pot. The remaining four seedlings per pot were cut 5 mm above and below the crown and plated on PDA amended with antibiotics to re-isolate pathogens. *Fusarium* isolates were identified based on morphology five days after plating. Remaining plants were monitored for above-ground symptoms and FRCR was rated at harvest using the disease-rating scale as described previously. Number of tillers, number of nodes, number of heads or pods, number of seeds, seed weight, plant height, and above-ground biomass data were collected to assess crop quality.

### 2.6. Statistical Analysis

All statistical analyses were conducted using JMP 17 (SAS Institute) unless stated otherwise. Choices of cover crop effects on FRCR disease severity in barley and soybean were tested using mixed linear models with the restricted maximum likelihood (REML) method and post hoc Tukey’s HSD test (*p*-value ≤ 0.05), with replicates and trial considered as random effects. Correlation between FRCR in each cash crop and bacterial functional group was measured using Pearson’s correlation with significance defined as *p*-value ≤ 0.05. Effects on root isolates were tested using GLIMMIX with Log-link Poisson distribution and post hoc Tukey’s HSD test. Means for the small greenhouse trial, testing the pathogenicity of a selection of different isolates, were tested using Kruskal–Wallis and Dunn’s test (package: “dunn.test”) on R. Mean values from the soil treatment and pathogen load greenhouse trial were also tested using mixed linear models with the REML method, with pots planted in replicated blocks considered as random effects. Principal component analysis was carried out to compare agronomic data and disease severity from the greenhouse trial.

## 3. Results

The results presented include a summary of amplicon sequencing of cover crop residue and OTU clustering; differential abundance analysis; a visual rating of FRCR in soybean and barley in the field; pathogen isolation and phylogenetic analysis; assays testing *Fusarium* spp. pathogenicity; and greenhouse trials assessing the disease-suppressive effects of cover crop soils.

### 3.1. Sequencing and OTU Clustering

**Figure 1:**
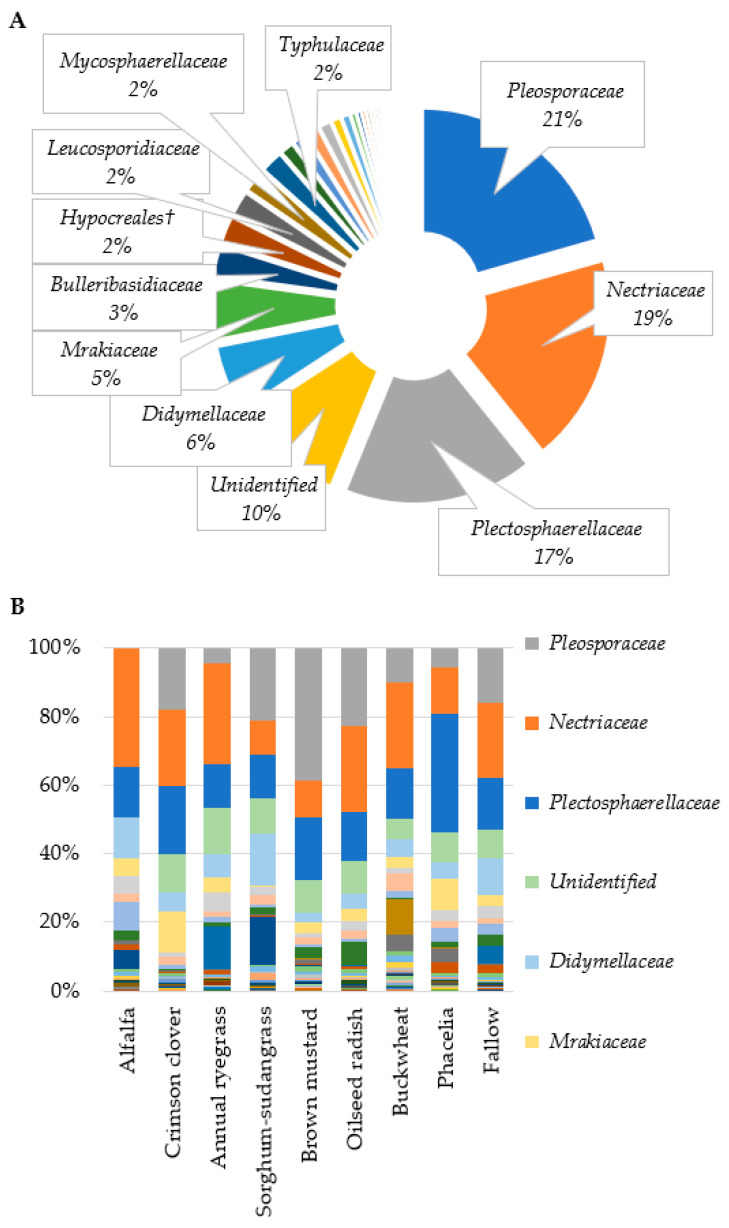
Fungal family relative abundance profiles in residue samples. (**A**) Pie chart summarizing the average relative abundance of these families across 54, n = 54; (**B**) differences in relative abundance of fungal families by cover crop, n = 6. † represents enigmatic taxa within the highlighted order.

From the 56 samples, ITS1 sequencing yielded 5,891,123 reads. Post-trimming and chimera removal, the sequence count was refined to 4,924,314 clean reads. These were further clustered into 8588 OTUs. Subsequent filtering eliminated unclassified and low-abundance OTUs, leaving 898 OTUs for analysis (Table S1). Among these, 462 OTUs were identified using a reference database, while 436 were annotated de novo (Table 2). The top three most abundant fungal taxa identified at the family level were *Pleosporaceae*, *Nectriaceae*, and *Plectosphaerellaceae* (Figure 1A). The influence of cover crops on the abundance of fungal OTUs was observable even at the family level (Figure 1B).

**Figure 2:**
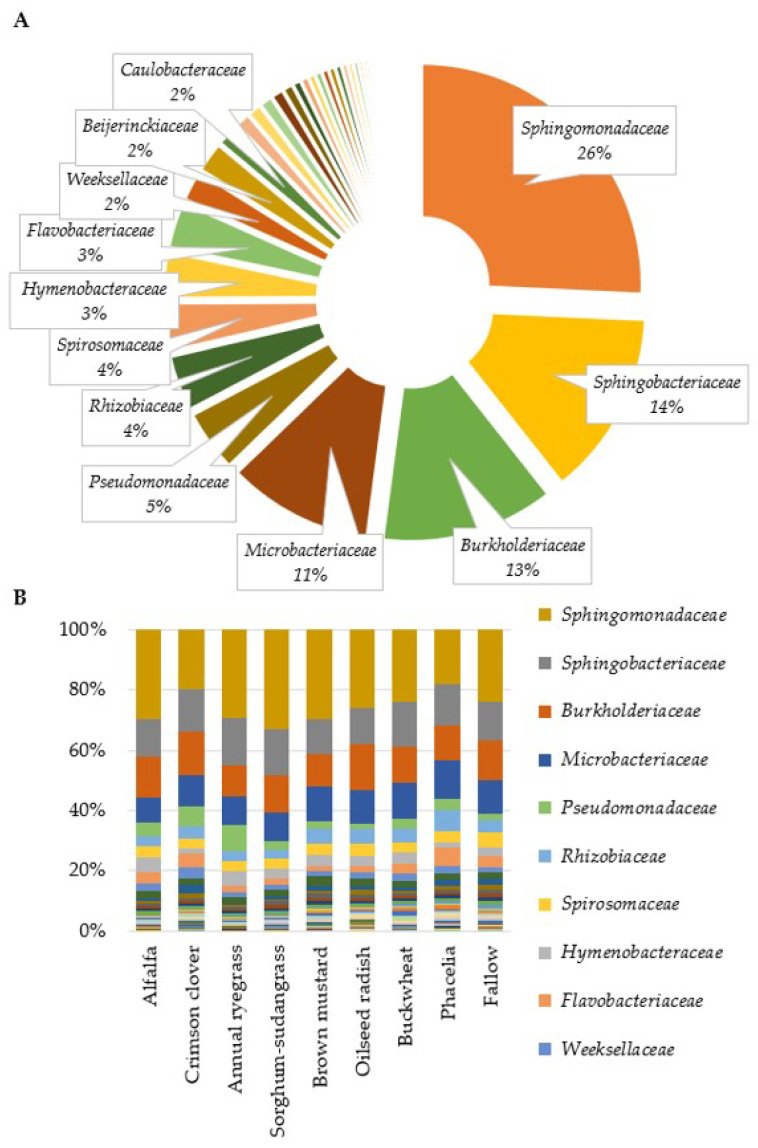
Bacterial family relative abundance profiles in residue samples. (**A**) All-sample combined mean abundance of bacterial families, n = 54; (**B**) differences in relative abundance of bacterial families by cover crop.

The sequencing of the 16S rRNA gene for bacterial communities yielded 189,923 reads, narrowed down to 174,085 non-chimeric, filtered reads. These were then categorized into 17,170 OTUs. Post-abundance and taxonomy filtering, 1067 OTUs remained, with 765 identified from a reference database and 302 annotated de novo (Table S2). The most prevalent bacterial family in the residue was *Sphingomonadaceae*, succeeded by *Sphingobacteriaceae* and *Burkholderiaceae* (Figure 2A). Additionally, the composition of bacterial families varied with the type of cover crop used (Figure 2B).

### 3.2. Fusarium Abundance in Soil and Residue

The PERMANOVA results indicated that the choice of cover crop had a significant effect on fungal community composition in the residue collected from the first trial (*p* < 0.01) but not the second (*p* = 0.41). Overall, fungal community composition in annual ryegrass was significantly different from all other cover crop treatments except fallow (Table 3). The fungal community associated with phacelia was also significantly different from alfalfa, sorghum-sudangrass, and buckwheat. The composition of bacterial communities associated with annual ryegrass displayed significant differences compared to all other cover crop treatments, with the exception of sorghum-sudangrass. The alfalfa bacterial community was similar to all other cover crop treatments. The unmanaged fallow control had a bacterial community composition distinct from annual ryegrass, sorghum-sudangrass, and buckwheat. With phacelia, the bacterial community was also different from sorghum-sudangrass, brown mustard, and buckwheat. The buckwheat bacterial community was also different from that of crimson clover and oilseed radish. The oilseed radish bacterial community was also different from sorghum-sudangrass. The brown mustard bacterial community was also different from crimson clover. Finally, the sorghum-sudangrass bacterial community was also distinct from that of crimson clover.

**Figure 3:**
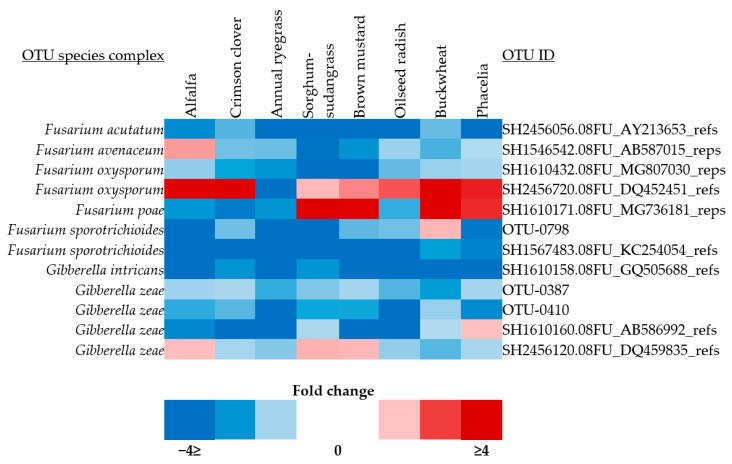
Differentially abundant *Fusarium* OTUs in cover crop residue. The heatmap shows the fold change in the relative abundance of *Fusarium* OTUs from each cover crop residue compared to fallow residue. Species names are listed on the left and OTU IDs are listed on the right.

A higher abundance of *Fusarium* OTUs was found in the residue collected from all plots in the second trial. In the data from both trials combined, 402 residue fungal OTUs were identified as pathotroph, 185 as saprotroph, and 8 as symbiotroph. The most abundant fungal pathotrophs were *Alternaria* spp., *Plectosphaerella* spp., *Colletotrichum* spp., and *Fusarium* spp. A total of 12 *Fusarium* spp. were differentially abundant by cover crop (Figure 3). *F. oxysporum* and *F. poae* were the only OTUs which significantly increased in abundance as a response to growing a certain cover crop when compared to the unmanaged fallow control. The *F. oxysporum* OTU was higher in abundance compared to fallow in every cover crop except annual ryegrass, in which the opposite trend was observed. The *F. poae* OTU was higher in sorghum-sudangrass, brown mustard, buckwheat, and phacelia residue compared to fallow.

### 3.3. FRCR in Soybean and Barley in the Field

**Figure 4:**
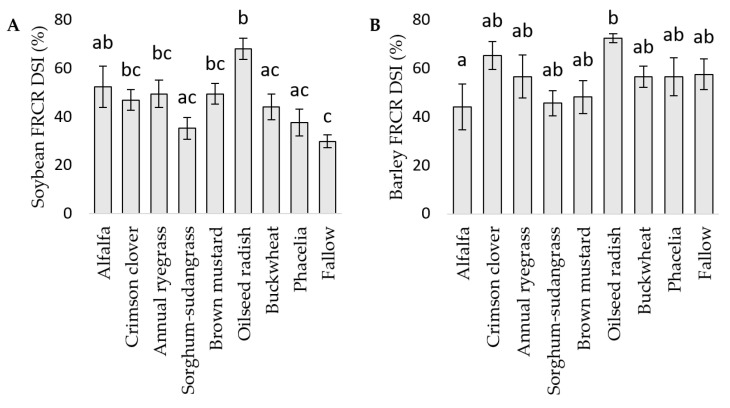
Effects of previously planted cover crops on soybean (**A**) and barley (**B**). Fusarium root and crown rot (FRCR) disease severity index (%) with data from 2019 and 2020; n = 6. Different letters represent statistically significant differences at *p*-value < 0.05.

The choice of cover crop significantly influenced FRCR in soybean. Soybean planted after oilseed radish had significantly high FRCR severity compared to soybean after buckwheat, phacelia, sorghum-sudangrass, and the fallow control (Figure 4A). Alfalfa, in the first trial, was associated with relatively high FRCR in soybean, but the opposite trend was observed in the second trial. Meanwhile, oilseed radish was associated with relatively high FRCR and sorghum-sudangrass was associated with low FRCR in both trials. FRCR in barley were observed in high levels in all crops, with an average DSI greater than 40%, and cover crop identity still significantly influenced the disease severity. Barley grown after alfalfa had significantly lower FRCR compared to barley grown after oilseed radish (Figure 4B). Barley grown after annual ryegrass was associated with high FRCR in the first trial but low FRCR in the second trial, and barley grown after alfalfa had the opposite effect. Similar to the observations with soybeans, FRCR in barley were consistently high when grown after oilseed radish and relatively low after sorghum-sudangrass in both trials.

Certain bacterial functional groups, including “ureolysis”, “animal parasites or symbionts”, and “human-associated”, were linked with barley and soybean FRCR DSI (Table 4). Interestingly, the “plant pathogen” functional group was not significantly associated with barley or soybean disease. However, this group only listed two OTUs, which were in relatively low abundance. Furthermore, several of the bacterial families listed in the other functional groups included plant pathogenic species.

### 3.4. Pathogen Isolation and Phylogenetic Analysis

**Figure 5:**
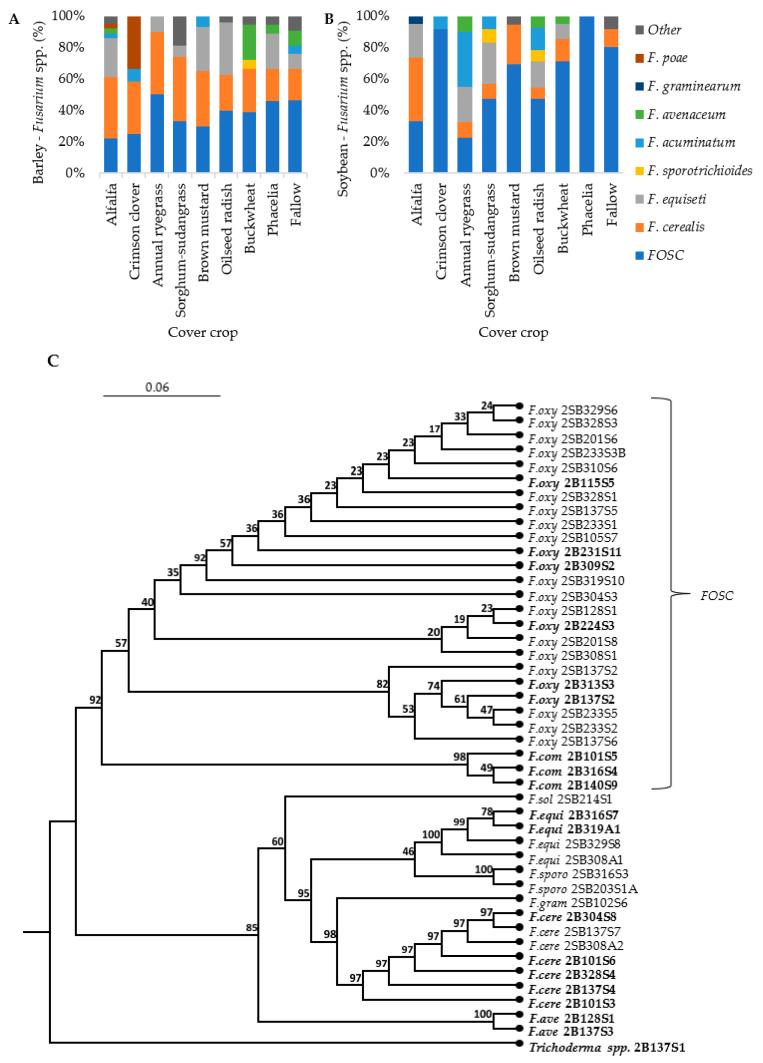
*Fusarium* isolates from barley and soybean roots collected from the second trial. Proportions of *Fusarium* spp. isolates by the total number of *Fusarium* isolates per cover crop, for barley (**A**) and soybean (**B**), presented in stacked bar graphs (n = 6). A rooted phylogenetic tree cladogram with sequences from the eight main *Fusarium* spp. isolates and a *Trichoderma* spp. Tef-1α sequence set as the outgroup (**C**); n = 45. The labels list a short form of the species name along with the unique isolate ID, with barley isolates in bold. *F.oxy* is short for *F. oxysporum*, *F.com* = *F. commune*, *F.sol* = *F. solani, F.equi* = *F. equiseti*, *F.sporo* = *F. sporotrichioides*, *F.gram* = *F. graminearum, F.cere* = *F. cerealis*, and *F.ave* = *F. avenaceum. FOSC = F. oxysporum* species complex.

*F. oxysporum* was the pathogen most commonly isolated from barley and soybean roots. A total of 379 root isolates were identified as *Fusarium* spp., with 227 from barley roots and 152 from soybean roots. Isolates were identified as *F. oxysporum* species complex (FOSC), *F. cerealis*, *F. equiseti*, *F. graminearum*, *F. avenaceum*, *F. acuminatum* (Ellis and Everhart; teleomorph: *Gibberella acuminata* (Wollenw.)), *F. poae* (Peck (Wollenw.)), and *F. sporotrichioides*. *F. cerealis*, FOSC, and *F. equiseti* were the most abundant isolates from barley roots, making up 33%, 31%, and 19% of all *Fusarium* isolated from barley, respectively (Figure 5A). FOSC and *F. cerealis* were the most abundant isolates from soybean, comprising 63% and 16% of the *Fusarium* isolated, respectively (Figure 5B). The previous crop did not have an effect on the number of *Fusarium* isolates or any of the individual species. The highest numbers of *Fusarium* spp. were isolated from barley and soybean planted after brown mustard. The phylogenetic tree indicated that there were three *F. oxysporum* branches, which were closely related to *F. commune* isolates. *F. graminearum* isolates were closely related to *F. cerealis* and the *F. sporotrichioides* isolates were closely related to *F. equiseti* (Figure 5C).

### 3.5. Fusarium Inoculum Virulence Assay

**Figure 6:**
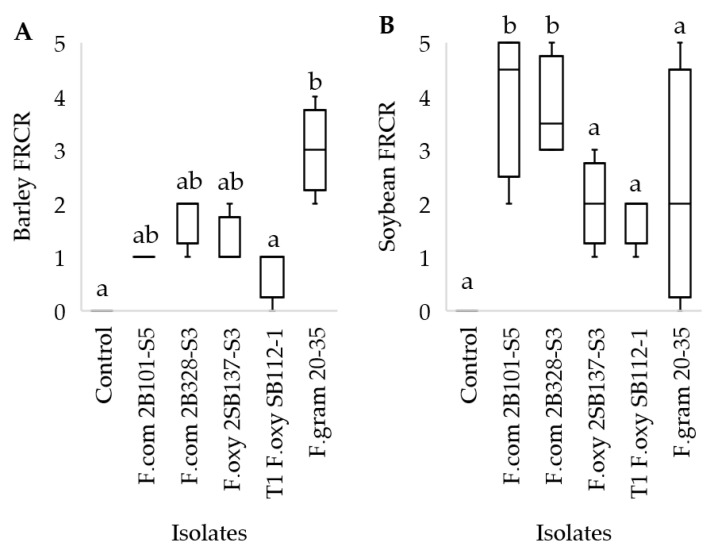
Box plots representing the effect of various field isolates used as *Fusarium* inoculum on FRCR in barley (**A**) and soybean (**B**), based on the 0 to 5 disease-rating scale. The whiskers represent the upper and lower 25% quartiles and the box represents 50%. *F.oxy* is short for *F. oxysporum*, *F.com* = *F. commune*, and *F.gram* = *F. graminearum,* all compared to non-inoculated control. Connecting letters represent significant differences *p* < 0.05.

A selection of field isolates, including *F. oxysporum*, *F. commune*, and *F. graminearum*, tested for virulence against soybean and barley in the greenhouse were found to cause disease (Figure 6).

Under controlled conditions, the *F. commune* isolates were found to be most virulent in soybean, even though they were isolated from barley roots; meanwhile, *F. graminearum* was found to be most virulent in barley. These isolates were chosen for testing the disease-suppressive effects of cover crop soils. However, in the greenhouse experiment with field soil, the soybean isolate did not see disease comparable to field conditions. As such, the subsequent greenhouse experiment was conducted only on barley. *F. graminearum* spring wheat spike isolate 20–35 [44] was used as the pathogen inoculum in the subsequent experiment, as the *F. graminearum* isolate from this study was lost to contamination.

### 3.6. Disease-Suppressive Effects of Cover Crop Soils

**Figure 7:**
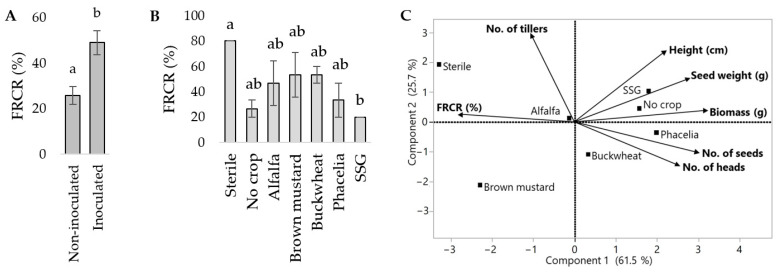
The effect of *F. graminearum* inoculation on FRCR DSI in barley in the greenhouse trial (**A**). The effect of different cover crop soils on FRCR DSI in barley under high disease pressure (**B**). Connecting letters represent significant differences *p* < 0.05. Principal component analysis comparing disease severity and agronomic data collected from barley greenhouse trials, with *F. graminearum* inoculum (**C**). FRCR = Fusarium root and crown rot; SSG = sorghum-sudangrass.

*F. graminearum* was re-isolated from all destructively sampled roots collected early in the greenhouse trial. Cover crop soils differentially influenced FRCR in barley based on visual symptoms assessed at harvest. Pathogen inoculation significantly increased FRCR severity (Figure 7A). Plants growing in the brown mustard and alfalfa soils had relatively high FRCR disease severity, while barley in the no-crop and sorghum-sudangrass soils had relatively low disease severity regardless of inoculum. Barley grown in phacelia soil produced the most seeds, whereas barley grown in brown mustard soil produced the least seeds, regardless of inoculum. Barley grown in sorghum-sudangrass soil was significantly lower compared to sterile soil (Figure 7B). The effect of cover crop soils, as well as the interaction effect of cover crop by inoculum, on the seed weight was significant. The seed weight from barley grown in “no-crop” soil (weed-free and tilled) was significantly higher than that from brown mustard soil, regardless of inoculum. Barley grown in phacelia and sorghum-sudangrass soils with inoculum had a higher seed yield than those without inoculum. The first two components of the PCA, comparing agronomic measurements and FRCR severity in the inoculated treatment, represented more than 80% of the variance (Figure 7C). FRCR were negatively correlated with all other agronomic measurements except for number of tillers. FRCR were strongly associated with brown mustard soil, while they were negatively associating with phacelia, sorghum-sudangrass, and no-crop soils.

## 4. Discussion

The choice of cover crop influenced FRCR severity in subsequent crops, both by changing the *Fusarium* abundance and species diversity in the residue but also by creating more, or less, disease-suppressive soil microbiomes. High levels of FRCR were observed in both barley and soybean for the field trials and the disease severity was influenced by the previously planted cover crop. Considering that the FRCR severity did not directly correlate with *Fusarium* abundance, it is likely that barley and soybean were under similar disease pressures in all plots and, thus, the difference in disease is attributed to the disease-suppressive soils associated with specific cover crops. Previously, we found that the soil microbiome associated with specific cover crops included some beneficial bacteria and fungi, which aids in plant resilience against FRCR [13]. Barley and soybean planted after oilseed radish had the overall highest observable FRCR symptoms. Both field and greenhouse trials indicated that sorghum-sudangrass was associated with lower FRCR in barley. This research, conducted in PE, Canada, provides valuable insights into the influence of cover crop-associated microbiomes on FRCR, but while findings are immediately relevant to similar temperate maritime environments, their applicability to other geolocations may depend on factors such as climate similarity, soil characteristics, and prevalent *Fusarium* species.

Certain bacterial functional groups were associated with barley and soybean FRCR, including families which have plant pathogenic species. Therefore, the positive association of these populations with barley and soybean disease may indicate that certain bacterial pathogens were contributing to root disease. This highlights the need to improve the FAPROTAX databases to better distinguish between bacterial functional groups that have ecological relevance.

Higher FRCR in barley and soybean were associated with increased *Fusarium* load in the residue. Different *Fusarium* spp. were identified as dominant causal agents of disease in the two crops. Other pathogens, including *Bipolaris sorokiniana* ((Sacc.) Shoemaker; teleomorph: *Cochliobolus sativus* (Ito and Kuribayashi) Drechs. ex Dastur), were also isolated (however, less frequently). Therefore, it is possible that not all the disease observed was caused by *Fusarium* spp. However, most of the symptoms were caused by the *Fusarium* spp., as they were the most commonly isolated pathogens from barley and soybean roots. As different cash crops host different *Fusarium* spp. as major pathogens, the choice of cover crops should reflect their impact on individual *Fusarium* spp. in the soil. For example, *F. oxysporum* was the more abundant pathogen isolated from soybean, while *F. cerealis*, the *F. oxysporum* species complex (FOSC), and *F. equiseti* were commonly isolated from barley. *F. oxysporum* is a common soybean pathogen, but is less frequently associated with FRCR disease in barley [45,46].

Interestingly, many of the barley isolates that were identified as FOSC were *F. commune*, which was only distinguishable from *F. oxysporum* using the Tef1-α primer set but not the ITS primer set. *F. commune* was recently identified as the causal agent of FRCR in field peas in the Maritime region of Canada [47]. It was also previously reported to cause disease in soybeans in Alberta [43]. However, to the best of our knowledge, this study is the first report of *F. commune* causing root disease in barley in Canada.

Oilseed radish and brown mustard produce isothiocyanates (ITC), which are known to have toxic effects against soil-borne microorganisms [48]. However, these crops were associated with high FRCR in both barley and soybean, likely because the most commonly isolated pathogen was *F. oxysporum*. Smolinska et al. [48] found that *F. oxysporum* was more resistant to ITCs, with the inhibition of spore germination observed only in the presence of a higher concentration of certain ITCs, such as ethyl, benzyl, and phenethyl. Furthermore, the biofumigant effects of brassicas are less successful under no-till conditions. In our greenhouse trial, brown mustard soil led to an increase in FRCR in barley. This is likely because the residue was mixed with the soil, leading to the biofumigation of beneficial microorganisms, while having little effect on the *Fusarium* spp. that was highly abundant even in the soil. This is similar to findings reported by Nallanchakravarthula et al. [49].

*Fusarium* spp. are ubiquitous and difficult to eradicate; however, different management practices can be used to suppress certain pathogenic species or promote *Fusarium*-suppressive soil. These approaches can include the secretion of toxic metabolites and increasing the abundance of beneficial soil microbes [50]. Sorghum-sudangrass, in our study, was correlated with less FRCR in both barley and soybean in the field. Sorghum-sudangrass is known to produce p-hydroxybenzoic acid, which has anti-fungal effects against *F. oxysporum* [51]. Previously, sorghum-sudangrass was also found to increase the abundance of symbiotrophic fungi, including important arbuscular mycorrhizal fungi [13]. As such, our results show that sorghum-sudangrass, when used as cover crop in no-till systems, may have disease-suppressive effects against FRCR in subsequently planted crops. It is possible that sorghum-sudangrass altered the structure of the soil microbiome, thereby conferring disease-suppressive abilities to the associated soil. We have shown that sorghum-sudangrass can suppress *Fusarium* spp. in the soil; however, it was not clear whether it suppresses other pathogens such as *Pythium* spp. and *Rhizoctonia* spp., which were isolated from samples collected early in the growing season [results not shown]. Further research with on-farm data with different field histories would be required to better understand the underlying mechanisms involved in disease suppression by sorghum-sudangrass soil. In our greenhouse trial, *F. graminearum* inoculum caused significantly more disease in the sterile soil, indicating that the legacy microbial community from any cover crop played a role in mitigating the damaging effects of high pathogen load, thus making the soil more resilient. The “no crop” soil treatment was consistently associated with low FRCR in both barley and soybean, even with pathogen inoculum. It is possible that leaving fields undisturbed where no crops were planted enhanced soil stability, promoted weed diversity, and caused subsequent changes in the microbial community that led to disease-suppressive soils [5].

The pathogen load in cover crop residue in no-till cropping systems plays an important role in root disease development [52]. Differential abundance analysis identified several pathogenic fungal OTUs that were affected by cover crop identity, including 12 *Fusarium* OTUs. *F. oxysporum* and *F. poae* were more abundant in cover crop residue compared to fallow residue. The differences in the fungal community composition in cover crop residue may be attributed to differences in residue structure, both above and below ground. The amount of plant material and the particle size of the residue changes the rates of decomposition, thereby creating different microenvironments for the associated microorganisms [4]. Furthermore, *F. graminearum*, as well as other *Fusarium* spp., are known to cause Fusarium head blight (FHB) in crops such as barley and wheat [5,53,54]. Increased pathogen load in the residue may increase FHB incidence and severity, as *Fusarium* resting structures are known to shoot spores high into the air, leading to the infection of aboveground plant parts [5,54]. This may indicate that cover crop residues with high *Fusarium* abundance may increase FHB incidence in subsequently planted crops. *F. graminearum* was detected in the field isolates; however, it was not the most abundant pathogen. FHB was not detected in either field trial, likely also because the weather conditions were not conducive for this disease. Studying the effects of certain cover crops on pathogen complexes involved in early season disease, as well as FHB, is critical in better understanding their impacts on plant production.

## 5. Conclusions

The results from this study clearly indicate that FRCR in both barley and soybean are highly influenced by antecedent cover crop residue. This effect is related not only to pathogen populations, but also to changes in the overall microbial composition in both soil and plant residue. We have shown that crop residue can host several fungal plant pathogens, which play an important role in causing disease in subsequently planted crops in a no-till cropping system. In this study, *Fusarium* spp. were amongst the most abundant pathogens in the residue and were identified as the causal agents of FRCR in both barley and soybean. Crops planted after oilseed radish had significantly higher incidence of FRCR, while crops planted after sorghum-sudangrass had significantly lower FRCR incidence. This indicates that the soil microbiome associated with sorghum-sudangrass has disease-suppressive effects against FRCR. This study adds to the broader understanding of soil and plant residue microbiome effects on FRCR in barley and soybean with a community-level assessment of microbial interactions. The results from this study provide a solid basis for future research into the interactions between cover crop identity and disease suppressiveness. They may also be used as a basis for farmers to make management decisions for more sustainable agricultural practices.

## Figures and Tables

**Table 1:** Primer sequences and PCR conditions for taxonomic identification of *Fusarium* isolates.

Primer	Sequence (5′-3′)	Locus	Conditions
ITS1F *	CTT GGT CAT TTA GAG GAA GTA A	ITS1	5 min—95 °C ----40 cycles---- Denaturation: 30 s—94 °C Annealing: 30 s—52 °C Elongation: 1 min—72 °C 8 min—72 °C
ITS4 *	TCCTCCGCTTATTGATATGC		
EF1 **	ATG GGT AAG GA(A/G) GAC AAG AC	TEF-1α	8 min—95 °C -----35 cycles----- Denaturation: 30 s—95 °C Annealing: 60 s—53 °C Elongation: 1 min—72 °C 5 min—72 °C
EF2 **	GGA (G/A)GT ACC AGT (G/C)AT CAT GTT		

* ITS1 primers were designed by White et al. [38]. ** Tef-1α primers were designed by O’Donnell et al. [39].

**Table 2:** Summary of sequencing results and OTU clustering. Values in parentheses are percentages of total sequenced reads.

		Bacteria	Fungi
	Sequencing Platform	PacBio Sequel	Illumina MiSeq
	Target region	Full 16S rRNA	ITS1
	Number of samples	54	54
	Targeted reads per sample	5000	90,000
Raw data	Total nucleotides in data sets	286,168,882	2,945,618,500
	Total reads in data sets	193,619	11,782,472
	Mean read length	1478 bp	250 bp
	Mean reads per sample	3586	218,193
Quality control	Total reads after trimming	189,923 (98.1%)	5,891,123 (99.9%) *
	Post trimming mean reads	3517	218,192
OTU Clustering	Reads in OTUs	174,085 (89.9%)	4,924,314 (83.6%)
	Total predicted OTUs	17,170	8588
	Mean OTU length	1454 bp	282 bp
Filtered OTUs	Reads in OTU after filtering	130,412 (67.4%)	4,217,277 (71.6%)
	Total OTUs after filtering	1067	898

* Illumina paired end reads were merged before OTU clustering.

**Table 3:** Microbial community composition differences by cover crop pairs, measured using PERMANOVA. Fungal data are highlighted in yellow and bacterial data are highlighted in blue.

	Alfalfa	Crimson Clover	Annual Ryegrass	Sorghum-Sudangrass	Brown Mustard	Oilseed Radish	Buckwheat	Phacelia	Fallow
Alfalfa	-	NS	NS	NS	NS	NS	NS	NS	NS
Crimson clover	NS	-	0.04	0.03	0.05	NS	0.03	NS	NS
Annual ryegrass	NS	NS	-	NS	0.03	0.01	0.02	0.01	0.02
Sorghum-sudangrass	NS	NS	0.02	-	NS	0.05	NS	0.01	0.03
Brown mustard	NS	NS	0.02	NS	-	NS	NS	0.03	NS
Oilseed radish	NS	NS	0.01	NS	NS	-	0.05	NS	NS
Buckwheat	NS	NS	0.02	NS	NS	NS	-	0.04	0.03
Phacelia	0.05	NS	0.01	0.01	NS	NS	0.05	-	NS
Fallow	NS	NS	NS	NS	NS	NS	NS	NS	-

NS = not significant at *p* ≤ 0.05.

**Table 4:** A description of the bacterial functional groups associated with barley or soybean disease, including the families that are categorized under these groups according to the FAPROTAX database. Numbers indicate Pearson’s correlation coefficient. NS indicates not significant at *p* < 0.05.

Functional Group	Family	Barley FRCR	Soybean FRCR
plant pathogen	*Enterobacteriaceae*	NS	NS
	*Pseudomonadaceae*		
ureolysis	*Isosphaeraceae*	NS	0.5418
	*Acetobacteraceae*		
	*Beijerinckiaceae*		
	*Rhizobiaceae*		
	*Burkholderiaceae*		
	*Methylophilaceae*		
	*Pseudomonadaceae*		
	*Xanthomonadaceae*		
animal parasites or symbionts	*Acetobacteraceae*	0.3263	NS
	*Enterobacteriaceae*		
	*Pseudomonadaceae*		
	*Xanthomonadaceae*		
human associated	*Acetobacteraceae*	0.3079	NS
	*Enterobacteriaceae*		
	*Xanthomonadaceae*		

## Data Availability

Metagenomic sequencing data are available at NCBI BioProject: PRJNA781550.

## Supplementary Materials

The following supporting information can be downloaded at https://www.mdpi.com/article/10.3390/microorganisms12020404/s1, Table S1: Fungal OTU table; Table S2: Bacterial OTU table.
